# Low Diversity Bacterial Community and the Trapping Activity of Metabolites from Cultivable Bacteria Species in the Female Reproductive System of the Oriental Fruit Fly, *Bactrocera dorsalis* Hendel (Diptera: Tephritidae)

**DOI:** 10.3390/ijms13056266

**Published:** 2012-05-21

**Authors:** Zhanghong Shi, Lili Wang, Hongyu Zhang

**Affiliations:** State Key Laboratory of Agricultural Microbiology, Institute of Urban and Horticultural Pests, Hubei Key Laboratory of Insect Resource Application and Sustainable Pest Control, College of Plant Science and Technology, Huazhong Agricultural University, Wuhan 430070, China; E-Mails: shizh@mail.hzau.edu.cn (Z.S.); lilywang321@163.com (L.W.)

**Keywords:** bacteria, symbiosis, microbiota, *Enterobacteriaceae*, *Klebsiella oxytoca*

## Abstract

Our goal was to identify the bacteria inhabiting the reproductive system of the female oriental fruit fly, *Bactrocera dorsalis* (Hendel), and evaluate the chemotaxis of *B. dorsalis* to the metabolites produced by the bacteria. Based on 16S rRNA-based polymerase chain reaction-denaturing gradient gel electrophoresis (PCR-DGGE), 18 operational taxonomic units (OTUs) were assigned to the five bacterial classes *Betaproteobacteria*, *Alphaproteobacteria*, *Gammaproteobacteria*, *Bacilli* and *Actinobacteria*. Nine OTUs were assigned to *Gammaproteobacteria*, which was the most highly represented class. *Enterobacteriaceae* constituted the dominant family, and within this family, three genera and five species were identified, including *Enterobacter sakazakii*, *Klebsiella oxytoca*, *Klebsiella pneumoniae*, *Raoultella terrigena* and *Enterobacter amnigenus*. In this set, the first two species were the dominant components, and the latter three species were the minor ones. Finally, we found that the metabolites produced by *R. terrigena*, *K. oxytoca* and *K. pneumoniae* were attractive to the *B. dorsalis* adults, and in field studies, *B. dorsalis* adults were most attracted to *K. oxytoca*. Collectively, our results suggest that the female reproductive system plays an important role in the transfer of enterobacteria from the gut to fruit. Our data may prompt the development of a female-targeted population control strategy for this fly.

## 1. Introduction

The oriental fruit fly, *Bactrocera dorsalis* (Hendel), is a destructive pest whose females lay eggs in the agricultural fruits of South East Asian and Pacific countries. The fly infests over 250 plant species, including economically important crops such as coffee, chili peppers, and carambola, peach, citrus, mandarin and mango fruit trees [1]. When the *B. dorsalis* larvae develop, the infested fruits rapidly deteriorate, leading to vast crop losses. Many pest-control strategies, such as methyl eugenol/protein baiting, the sterile insect technique and insecticide spraying, have been widely employed for decades, but due to the high fecundity and adaptability of this species, *B. dorsalis* is still not under effective control. The number of female flies has resulted in significant losses in the fruit industry; thus, a novel control technique targeting female flies is urgently needed.

Insects harbor diverse microorganisms in their intestinal tract and other tissues. Throughout long periods of co-evolution, microbes and their hosts have developed complex interactions that range from pathogenesis to obligate mutualism [2]. The commensal microbiota is vital to many physiological and homeostatic functions of their host [3]. Recent investigations revealed that gut bacteria enhanced their host’s stress resistance [4,5], improved social interactions [6], and formed a persistent but potentially beneficial infection in their host [7,8]; the research that deciphers the interactions between prokaryotic bacteria and multi-cellular animals is rapidly advancing. Better pest control methods could result from these studies.

The intestinal tracts of adult *B. dorsalis* and *Ceratitis capitata* fruit flies harbor a diverse bacterial community. Phylogenetic analyses revealed that both flies contained bacteria from the genera *Enterobacter*, *Klebsiella*, *Citrobacter*, *Pectobacterium*, *Pantoea* and *Serratia* [7,9]. Although gut facultative bacteria play important roles in insect development, little is known about the introduction of bacteria to the insect gut or the ensuing commensal relationship between bacteria and host. The medfly’s *C. capitata* enterobacterial community was transferred by the female parent to the chosen fruit during oviposition, and established and proliferated within the fruit, and subsequently maintained throughout the fly’s life [10]. However, the mechanism by which gut bacteria were delivered from the host to fruit is unknown. In this study, we described the bacterial community in the female *B. dorsalis* reproductive tract, and we evaluated the relationship between these microbiota and their hosts. A culture-independent method—16S rRNA-based polymerase chain reaction-denaturing gradient gel electrophoresis (PCR-DGGE)—was used to classify the bacteria species. Because bacteria release volatile components during their interactions with other organisms [11], we hypothesized that the fly gut microbiota produced chemoattractive metabolites. The chemoattraction between selected cultivable bacteria and *B. dorsalis* male and female adult flies was evaluated.

## 2. Results and Discussion

### 2.1. PCR-DGGE Analysis of the Bacterial Community in the *Bactrocera dorsalis* Female Reproductive System

**Figure 1:**
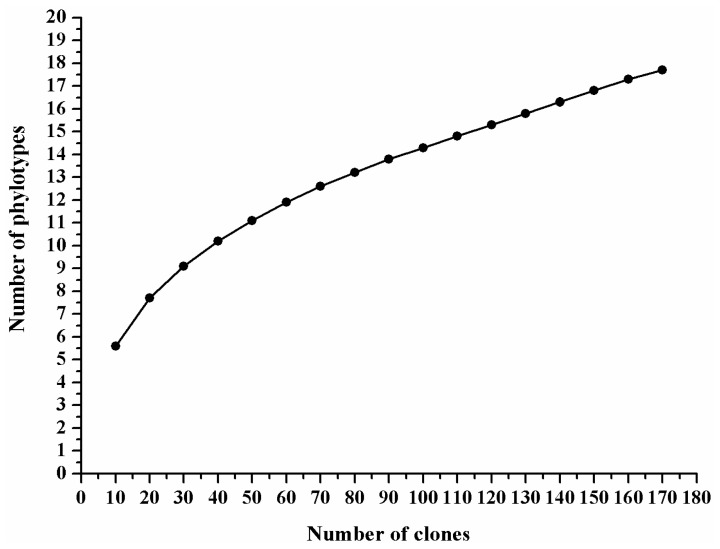
Rarefaction curves based on 16S rRNA gene clones generated from the reproductive system of female *Bactrocera dorsalis* adults.

Flattening of the rarefaction curve suggested that sampling was of adequate depth and that additional sampling would produce few additional operational taxonomic units (OTUs) (Figure 1). The 16S rRNA gene library contained sequences from 95.45% of the microorganisms in the reproductive system of female *B. dorsalis* adults. The Shannon and Simpson indices were 2.13 and 0.8399, respectively.

**Figure 2:**
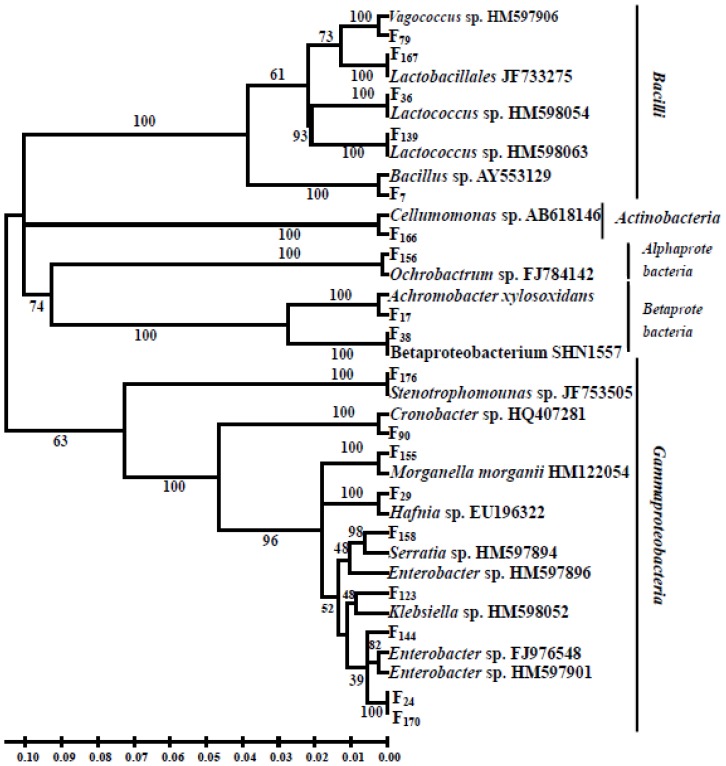
Phylogenetic tree of 16S rRNA gene sequences from bacteria in the adult female *Bactrocera dorsalis* reproductive tract. The tree was constructed using the neighbor-joining method. Sequences obtained in this study are presented as F_x_.

In total, 176 clones were obtained from the reproductive system of female *B dorsalis* adults. By PCR-DGGE analysis, the clones were grouped into 18 OTUs (Figure 2) and assigned into five bacteria classes: *Gammaproteobacteria* (9 OTUs), *Bacilli* (5 OTUs), *Betaproteobacteria* (2 OTUs), *Alphaproteobacteria* (1 OTU) and *Actinobacteria* (1 OTU). Sequences from *Gammaproteobacteria* were found in 142 clones, which represented 80.68% of our 16S rRNA library. This result suggested that *Gammaproteobacteria* dominated the bacterial community. The class *Bacilli* was also a major constituent, as it was represented by 30 clones (17.05%). Fewer sequences were classified into the classes *Betaproteobacteria*, *Alphaproteobacteria* and *Actinobacteria*.

**Figure 3:**
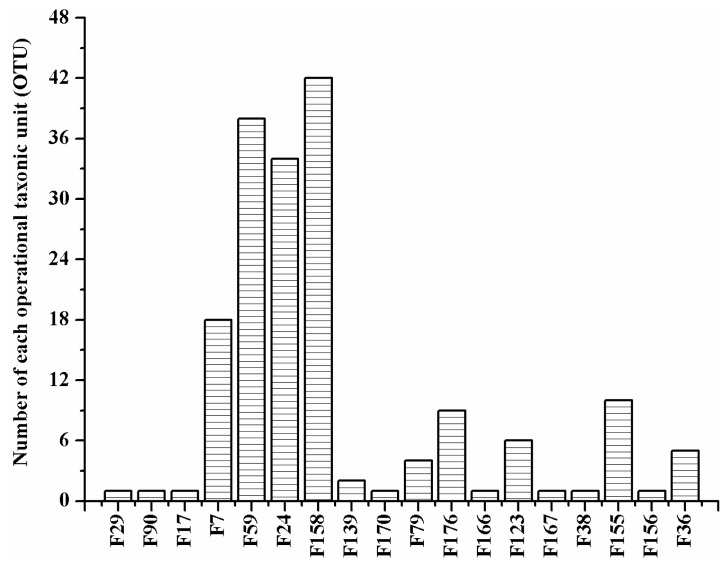
Species composition of the bacterial community in the adult female reproductive tract of *Bactrocera dorsalis*.

Four OTUs were considered predominant because they each constituted at least 10% of our library (Figure 3). These OTUs were F_158_ (23.86%), F_59_ (21.59%), F_24_ (19.32%) and F_7_ (10.23%), and they were affiliated with specific bacteria species. The first three OTUs were species within the family *Enterobacteriaceae*, while the last one belonged to *Bacillaceae*. Therefore, *Enterobacteriaceae* was the dominant family, with eight OTUs assigned into six genera. The OTU F_155_ represented 5–10% of our library. Only one clone corresponded to each of the following ten OTUs: F_29_, F_90_, F_17_, F_156_, F_167_, F_166_, F_38_, and F_170_. Thus, F_158_, F_59_, F_24_ and F_7_ were the major constituents of the bacterial community, while other OTUs were minor components at the species-specific level (Figure 3).

### 2.2. Cultivable Bacteria Species Identified from the Reproductive System of Female Adults

DGGE was used to screen the PCR products from 210 clones of cultivable bacteria species. Fourteen specific DNA fragments were identified and sequenced. By DOTUR analysis, these 14 sequences were grouped into five OTUs belonging to three genera—*Enterobacter*, *Klebsiella* and *Raoultella*—in the family *Enterobacteriaceae*. With the API-20E diagnostic kit, the five OTUs were identified as the following bacteria species: *Enterobacter sakazakii*, *Klebsiella oxytoca*, *K. pneumoniae*, *Raoultella terrigena* and *E. amnigenus*. The first two species were the dominant components, and the latter three were the minor ones.

### 2.3. The Trapping Activity of Metabolites from Three Species of Cultivable Bacteria Species

**Figure 4:**
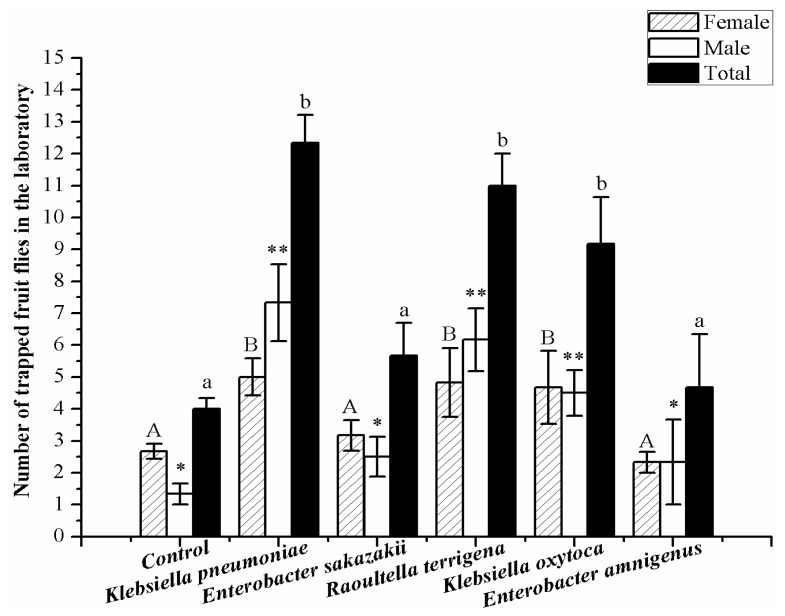
Trapping of *Bactrocera dorsalis* adults by metabolites produced by the cultivable bacteria. Columns represent the mean for each treatment. Different letters or stars above the standard error indicate significant differences between treatments, as determined by least-significant difference tests (*p* < 0.05).

Our bioassays demonstrated that metabolites produced by *Raoultella terrigena*, *Klebsiella oxytoca* and *K. pneumoniae* trapped a greater number of *B. dorsalis* adults than the controls (ANOVA: *F*_5, 27_ = 10.898, *p* < 0.001). *K. pneumoniae* trapped the greatest number of *B. dorsalis* adults, followed by *R. terrigena* and *K. oxytoca*. More female and male adults were trapped by *R. terrigena*, *K. pneumoniae* and *K. oxytoca* compared with other bacteria and the controls, and *B. dorsalis* male adults demonstrated the strongest behavioral responses to the metabolites from *K. pneumoniae* (*F*_5, 27_ = 9.380, *p* < 0.001; Figure 4).

**Figure 5:**
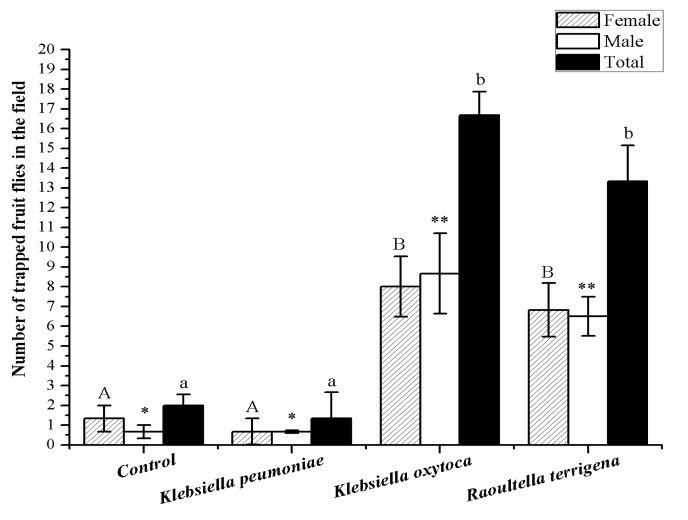
Field trapping assays of *Bactrocera dorsalis* adults with the metabolites from *Klebsiella pneumoniae*, *Klebsiella oxytoca* and *Raoultella terrigena*. Columns represent the mean for each treatment. Different letters or stars above the standard error bars indicate significant differences between treatments, as determined by least-significant difference tests (*p* < 0.05).

In the field bioassays, we found that the metabolites from *K. pneumoniae* caught a small number of fruit flies, which was the same result as the control experiments. Compared with the controls, markedly greater numbers of *B. dorsalis* adults were trapped by the other two bacteria species (Male: *F*_3, 11_ = 10.464, *p* < 0.001; Total number of trapped fruit flies: *F*_3, 11_ = 18.556, *p* < 0.001). *K. oxytoca* and *R. terrigena* trapped 8 ± 1.53 and 6 ± 1.35 female adults, respectively. Thus, there was a statistically significant increase in the number of adults trapped by these bacteria (*F*_3, 11_ = 7.058, *p* < 0.001; Figure 5).

### 2.4. Discussion

We identified a bacteria community consisting of the five classes *Betaproteobacteria*, *Alphaproteobacteria*, *Gammaproteobacteria*, *Bacilli* and *Actinobacteria* in the female reproductive system of *B. dorsalis* adults. The bacteria species classified as *Gammaproteobacteria* and *Bacilli* were the major constituents of this community, but other species were also present. Wang *et al*. [9] identified 55 OTUs from the intestinal tracts of laboratory-grown *B. dorsalis* adults, and the OTUs were assigned into the following seven classes: *Gammaproteobacteria*, *Actinobacteria*, *Bacteroidetes*, *Alphaproteobacteria*, *Deltaproteobacteria*, *Flavobacteria* and *Firmicutes*. Shannon and Simpson diversity indices of the *B. dorsalis* reproductive system bacteria community were significantly lower than those of the gut bacteria community. Therefore, a lower diversity bacteria community inhabits the female reproductive system of *B. dorsalis*. Compared with the *B. dorsalis* gut, the reproductive system is much more confined, and it has less contact with the external environment. Previous studies showed that the composition of the gut microbiota varied with the host’s diet [9], developmental stage [12] and physiological condition [13]. The distinct functions of the intestinal tract and the reproductive system may contribute to the difference in bacterial diversity in the two tissues.

The *Enterobacteriaceae* family, which was the dominant bacterial group in the gut of *B. dorsalis* [9] and *C. capitata* [7], also dominated the bacteria community inhabiting the reproductive system of *B. dorsalis*. New evidence suggests that the enterobacteria are transferred to fruit during oviposition; subsequently, the bacteria are passed to the fly offspring [12]. It is unclear how the enterobacteria are transmitted from the female gut to the chosen fruit during oviposition. Here, we confirmed that the female reproductive system of *B. dorsalis* also harbored a bacteria community that was dominated by *Enterobacteriaceae* bacteria, but this community was less diverse than that of the gut. These results suggested that the female reproductive system might bridge the pathway of enterobacteria transfer from the fly gut to the fruit. Thus, we postulate that the gut enterobacteria are dispersed into the female reproductive system, where they are subsequently transferred to the eggs, the fruit (during oviposition), and finally, the offspring.

Recent investigations have demonstrated that bacteria release their volatile molecules during their interactions with other organisms [11]. In the desert locust *Schistocerca gregaria*, the gut bacteria species *K. pneumoniae* and *E. cloacae* produce components of the locust cohesion pheromone [6]. Gut commensal bacteria influence mating preference by changing the levels of cuticular hydrocarbon sex pheromones in *Drosophila melangaster* [13]. Similarly, bacteria isolated from flies produce odors similar to the flies’ proteinaceous foods, and these odors attract flies [14]. In the present study, we presented evidence that the reproductive tract-associated bacteria *E. sakazakii*, *K. oxytoca* and *K. pneumoniae* released volatile metabolites that affected *B. dorsalis* adults’ behavior.

Ben-Ami *et al.* [15] demonstrated that the *C. capitata* gut population of *Klebsiella* species decreased significantly after fly sterilization; the addition of *K. oxytoca* to the post-irradiation diet markedly improved sterile male performance in copulation tests. *Klebsiella* spp. contributed to nitrogen fixation *in vivo* in the fly’s gut [10] and was associated with male mating success [16]. In our field assays, most *B. dorsalis* adults were trapped by the metabolites from *K. oxytoca*. Metabolites from *K. oxytoca* could be involved in the chemical communication between *B. dorsalis* individuals, and this potential new role for the bacteria enhances the insect-bacteria symbiosis. Taken together, *K. oxytoca* has an important function in both the gut and the female reproductive system. Although there have been some reports on the functional role of *K. oxytoca* [15], this role is only partially understood.

Male oriental fruit flies were strongly attracted by methyl eugenol (ME) [17]. ME is widely used as an attractant to control fruit flies. Although fewer *B. dorsalis* were attracted to the metabolites from the associated bacteria species here, these metabolites were different from ME. While ME attracted only males [17], metabolites produced by the cultivable bacteria of *B. dorsalis* trapped both female and males. In future studies, the bioactive chemicals in the metabolites of cultivable bacteria should be analyzed by gas chromatography-mass spectrometry (GC-MS) and high-performance liquid chromatography (HPLC). Elucidation of these chemicals would produce novel lead compounds for *B. dorsalis* traps. The different behavioral responses of *B. dorsalis* were documented by the laboratory and field bioassays in this study. For example, *B. dorsalis* exhibited strong chemotaxis to metabolites from *K. pneumoniae* only in the laboratory tests. This result suggested that some bioactive metabolites attract *B. dorsalis* from short-range distances only. Collectively, our results illustrate both the potential to develop a female-targeted strategy to control this polyphagous pest and the expansion of our understanding of insect-bacteria symbiosis.

## 3. Experimental Section

### 3.1. Origin and Dissection of *Bactrocera dorsalis*

All flies used in this study were from an established laboratory colony that originated from Guangzhou Province, China, and was cultured in the Institute of Urban and Horticultural Pests for at least 13 generations. The colony was maintained in cubical screen cages (0.3 × 0.3 × 0.3 m) with abundant food (sugar: yeast extract powder in a 3:1 ratio by weight) and water *ad libitum*. All experimental flies were maintained in the laboratory at 27 ± 1 °C, 70–80% r.h., and a L12:D12 photoperiod.

Virgin female adults were dissected after they reached an age of 11–14 days. Before dissection, female adults were anesthetized at −20 °C for 5 min. The fly cuticles were sterilized with 70% ethanol for 2 min and rinsed two times in sterile distilled water. Forty female adults were dissected aseptically with two pairs of sterilized tweezers in a plate containing 10 mL sterile distilled water, which was placed underneath a stereomicroscope. The forty intact reproductive systems, without outer ovipositor, were transferred into a tube containing 750 μL TE buffer [10 mmol·L^−1^ Tris-HCl (pH 8.0), 50 mmol·L^−1^ EDTA] and homogenized. The homogenate was used for total DNA extraction.

### 3.2. Total DNA extraction

To extract total DNA, the above homogenate was suspended in phosphate buffer, harvested by centrifugation, washed with the same buffer and dissolved in 557 μL TE buffer with 10 μL lysozyme (5 mg·mL^−1^). The mixture was incubated for 20 min at 37 °C. After 30 μL of 10% SDS and 3 μL of proteinase K (20 mg·mL^−1^) were added, samples were incubated for 40 min at 37 °C. Afterwards, 100 μL of NaCl (5 mol·L^−1^) and 80 μL of CTAB/NaCl were added, and the samples were incubated for 10 min at 65 °C. The samples were extracted with phenol/chloroform/isoamyl alcohol [25:24:1 (v/v/v)] and centrifuged for 4 min at 12,000 × g. Nucleic acids were precipitated with isopropyl alcohol, rinsed with 70% frozen ethanol and suspended in 30 μL TE.

### 3.3. PCR Amplification and Cloning of Bacterial 16S rRNA Genes

Bacteria-universal primers 968-GClamp (5′-CGCCCGCCGCGCGCGGCGGGCGGGGCGGGGG CACGGGGGGAACGCGAAGAACCTTAC-3′) and L1401 (5′-CGGTGTGTACAAGACCC-3′) were used for PCR amplification in this study [18,19]. Amplification was performed in triplicate in a 20 μL reaction system with a thermal cycler. To minimize PCR drift, several replicate PCR amplifications were combined [20]. Each reaction was composed of 14.6 μL ddH_2_O, 0.3 μL of each 10 μM each primer (working concentration of 0.15 μM), 2.0 μL 10× buffer (working concentration of 1× buffer), 1.2 μL 25 mM MgCl_2_ (working concentration of 1.5 mM), 0.4 μL 10 mM dNTPs (working concentration of 0.2 mM), 0.2 μL 1 U/μL DNA polymerase (working concentration of 0.01 U/μL) and 1 μL DNA template. PCR was run under the following conditions: 94 °C for 5 min, 30 cycles at 94 °C for 30 s, 56 °C for 30 s and 72 °C for 1 min, and a final extension period of 7 min at 72 °C. PCR products were run on an agarose gel (1.2% agarose, 1× TBE) and stained with ethidium bromide. Negative controls (no DNA added) were routinely included to check for contamination; no products were obtained from the controls. Bands were excised from the gel, and the DNA was purified with Axygen DNA purification kit (Axygen Biosciences, Inc., 33210 Central Avenue, Union City, CA, USA).

Purified PCR products were ligated into the pEASY-T_1_ Simple Cloning Vector with the pEASY-Blunt Simple Cloning Kit, using the manufacturer’s instructions (TRANS™, Beijing, China). Ligation products were transformed into *Escherichia coli* DH5α (Promega, Madison, WI, USA). Ampicillin-resistant transformants were selected by color-based recombination on Luria-Bertani (LB) agar plates containing ampicillin (100 μg·mL^−1^, Sigma, St. Louis, MO, USA), X-gal (20 mg·mL^−1^) and IPTG (40 mmol·L^−1^, Promega). One hundred and seventy-six transformants were picked randomly. Plasmid clones were examined by PCR with the vector-specific primer M13 and the primers used above (968-GC Clamp and L1401). These PCR products were used in the following DGGE analysis.

### 3.4. DGGE Screening of 16S rRNA Clones and Sequencing

DGGE was completed with a DCode™ Universal Mutation Detection System (Bio-Rad Lab., Hercules, CA, USA). For comparison between gels, the DGGE marker was from a selection of bacterial 16S rRNA gene products. PCR products were loaded onto 6% (w/v) polyacrylamide gels with a denaturing gradient ranging from 30 to 70% (100% corresponding to 7 mol L^−1^ of urea and 40% (w/v) deionized formamide in 0.5× TAE buffer). The reactions were run in 0.5× TAE buffer (pH 8.5) at a constant temperature of 60 °C with a voltage of 200 V for 10 min and 85 V for 16 h. After electrophoresis, the gels were stained with silver nitrate as described by Zhang and Jackson [21]. The DGGE bands were compared visually and grouped into different DGGE band profiles. One representative clone from each group was selected for sequencing. Bi-directional sequencing was performed with the vector-specific M13 forward and reverse primers by AuGCT Genscript Biotechnology Co., Ltd., Nanjing, China.

### 3.5. Phylogenetic Analysis of 16S rRNA for Phylotype Determination

Sequences were initially analyzed by DNAMAN software (version 6.0.3.93; Lynnon Corporation: St. Louis, MO, USA, 2007) and chimeric sequences were identified with the Chimera Detection tool of Ribosomal Database Project II release 10 (RDP-II) [22]. The remaining sequences were rapidly compared and aligned to known sequences in the GenBank database and RDP-II databases by Clustal X. DOTUR [23] was used to assign sequences to OTUs. Sequences that were in the same OTU_0.02_ (identity ≥ 98%) were considered to be from the same species [23]. Sequences that were in the same OTU_0.2_ (identity ≥ 80%) were considered to be from the same phylum [23]. BLASTN [24] was used to align sequences in OTUs to GenBank reference sequences. Phylogenetic trees were constructed using the neighbor-joining (NJ) algorithms in MEGA 4 software [25], and the accuracy of the tree topology was tested by 1000 bootstrap replicates [26].

### 3.6. Identification of Cultivable Bacteria Species by API-20E Diagnostic Kit

To investigate the growth profile of cultivable bacteria from the reproductive system of female adults, insects were surface-sterilized with 70% ethanol as before and dissected under aseptic conditions. According to the methods described by Behar *et al*. [12], three pools of 15 reproductive systems were homogenized in 750 μL TE buffer, serially diluted by 10^−4^–10^−8^, plated on LB agar and allowed to grow overnight at 37 °C. The representative bacteria colonies were selected by the morphology of each colony forming unit (CFU). Each representative bacteria colony was clonally propagated three times to ensure purity. Subsequently, each colony was cultured in LB medium at 37 °C for 48 h. To identify bacterial isolates, clonal cultures were prepared for 16S rRNA sequencing. The extraction and analysis of bacterial DNA, as well as bacterial isolate identification, were conducted as described above.

All bacteria isolates were Gram-stained and tested for oxidase/catalase activity. Only Gram-negative and rod-shaped bacteria were identified by the API-20E system (bioMérieux, Inc., Hazelwood, MO, USA). For each isolate, an API 20E strip was inoculated and incubated according to the manufacturer’s instructions. Bacterial species within *Enterobacteriaceae* were identified by the analytical profile indexes from the API-20E system. The ID profiles were rated from excellent to good, based on the API codes.

### 3.7. Fermentation and Bioassay

The bacteria culture and bioassays were performed as described by [27]. Briefly, 100 mL of LB medium were placed in 250 shaker flasks and autoclaved at 121 °C for 20 min. The flasks were cooled, and each flask was seeded with one of the five cultivable bacteria species. The flasks were placed in an incubated rotary shaker (37 °C, 200 rpm) for 144 h. To test for attraction of *B. dorsalis* adults, 10 μL of each species was transferred to a small cotton ball, which was placed into a fruit fly trapping apparatus. LB medium was used as the control. In our laboratory, the trapping activity was evaluated by cage-top bioassays on the flies. Three trapping apparatuses (cleaned with cotton balls dipped in 75% alcohol) were hung from the top of each cage (70 cm in diameter × 80 cm in height). Each experimental set-up was performed in triplicate. One hundred 14-day-old flies (50 females and 50 males) were released into the cage, which was covered by a thick black cloth. After 24 h, the number and gender of the trapped flies was recorded. The replicate experiments were conducted with new flies and new trapping apparatuses. All experiments were completed in the laboratory at 27 ± 1 °C and 70–80% r.h.

The field bioassays were conducted in the citrus orchard of Wuhan city, Hubei Province, in August 2010. The trapping apparatuses were suspended from randomly selected citrus trees with intervals of 20 m. The apparatus bottom was positioned 1.5 m above the ground. The field bioassays lasted for 30 days. Once every 3 days, the apparatuses were cleaned, the trapped flies were removed and counted, and the metabolites were reintroduced. Bacterial metabolites were tested by three traps at the same time, and twelve trapping apparatuses were used in the field bioassays.

### 3.8. Data Analysis

The data were normally distributed, as determined by the One-Sample Kolmogorov-Smirnov Test. Differences in the attraction of flies to metabolites from bacteria species were detected by ANOVA. To determine cohort group differences, the least-significant difference test served as a multiple comparison procedure. All tests were performed with the statistical software SPSS for Windows (version 12.0; SPSS Inc.: Chicago, IL, USA, 2003).

## 4. Conclusions

In this study, a bacterial community composed of *Betaproteobacteria*, *Alphaproteobacteria*, *Gammaproteobacteria*, *Bacilli* and *Actinobacteria* was discovered in the female reproductive system of *B. dorsalis. Gammaproteobacteria* was the most highly represented class. At the family-specific level, *Enterobacteriaceae* constituted the dominant population. Additionally, three genera and five species of cultivable bacteria belonging to *Enterobacteriaceae* were identified as *Enterobacter sakazakii*, *Klebsiella oxytoca*, *K. pneumoniae*, *Raoultella terrigena* and *E. amnigenus*. The first two species were the dominant components, and the latter three were the minor ones. Based on our results, we hypothesize the major bacterial species of *B. dorsalis* firstly disperse into the female reproductive system and then into fruit during oviposition whereby they are transmitted to the offspring. Finally, we found that *K. oxytoca*’s metabolites trapped the greatest number of *B. dorsalis* adults in the field. *R. terrigena* and *K. pneumonia* trapped fewer flies. Thus, our results indicate that there is the potential to develop a female-targeted control strategy for this fly.
